# Circular to the Fellows of the American Society of Dental Surgeons, and the Profession Generally

**Published:** 1848-04

**Authors:** C. O. Cone

**Affiliations:** Chairman of the Committee of Practical Dentistry. Baltimore


### Miscellaneous Notices.

*To the Fellows of the American Society of Dental Surgeons, and the Profession generally:*

Medicine and all its branches, has arose from mere manual assistance, to its scientific dispensation, by the accumulation of facts, gathered from deductions and experience. Dental surgery has long suffered as a profession, in both respectability and usefulness, from a concealment or retention of ascertained facts or improvements connected with the profession. This being known to the American Society of Dental Surgeons; at its last annual meeting, with a desire to carry out its avowed object of elevating and adding scientific knowledge to the profession, passed the following resolutions:

"*Resolved,* That the American Society of Dental Surgeons shall, at its annual meetings, appoint a committee of five of its active members, who shall be known as a Committee of Practical Dentistry, the duty of which shall be to make an annual report to the Society of the improvements effected in this country in the management of disease coming within the scope of the dental practitioner, and the condition and progress of dental knowledge in America during the year of their service."

"*And be it further resolved,* That it shall be the duty of each member of this Association, to communicate all information, or novel results in practice, at their earliest possible convenience to the above committee, that the above resolution may be more fully carried out."

To enable the committee appointed under the above resolutions, to fulfil the duty thus imposed on them, it is absolutely important that every member of the profession, who either respects his calling, his interest or himself, should contribute such information as he thinks may be rendered serviceable to the profession. There is hardly any one connected with the profession, that has not some peculiar method in his operations or practice, which he thinks superior to others generally known; an instrument or principle not in common use, or a case in practice which may illustrate some principle. Let all such be communicated, however trifling they may be, together with an explanation of their use and advantages, and should they be the agent in lessening only one pang of human suffering, their author would exhibit a philanthropic spirit.

The excuse by members of the profession, of a want of time, rendering it impossible to comply with the above, is an acknowledgement of selfishness and ingratitude, that no honorable practitioner should be guilty of. Must a profession do all for its members, when they withhold from it their support? Does a profession not only deserve, but does it not demand a return? And is this bestowal of time and energy on the profession cast away from individual interest? He that would bury within himself, or withhold from the profession an improvement or discovery in the minutest point of practice, from selfish motives, and individual interest, is unworthy of a place in, and unfit to associate with the society of the intelligent and scientific. From such we expect little, but from the liberal minded, the Association and the profession expect much. Every individual on entering the profession, becomes entitled to all its privileges and immunities, and also incurs an obligation to exert his best abilities to maintain not only its dignity and honor, but exalt its standing by extending the bounds of its usefulness.

It is hoped that this appeal will be answered by the profession generally, and particularly by the Fellows of the American Society of Dental Surgeons, by forwarding to the chairman of the committee, as early as the first of June next, such information as above referred to, or may come under the spirit of the resolutions.

It would hardly appear necessary to assure any that all contributions shall meet with just and careful considerations in the committee's report.

C. O. CONE,

*Chairman of the Committee of Practical Dentistry.*

BALTIMORE, *March* 14, 1848.

